# Supplementary material for: New job, new habits? A multilevel interrupted time series analysis of changes in diet, physical activity and sleep among young adults starting work for the first time
Source: Int J Behav Nutr Phys Act. 2025 Jan 28;22:10. doi: 10.1186/s12966-024-01682-8 (PMC11773725; doi:10.1186/s12966-024-01682-8)
Supplement: Supplementary file 1 — Supplementary Material 1: Supplementary Table 1: Likelihood Ratio Tests Description: Additional file 1: A table depicting likelihood ratio tests conducted to determine the best model fit for each outcome. [file 12966_2024_1682_MOESM1_ESM.pdf]

Supplementary Table 1: Likelihood ratio test for comparison with previous model

	Vegetables	Fruit	Physical activity	Sleep
Single-Level Model (m1)	ref	ref	ref	ref
Random intercept Model (m2)	<2.2e-16	<2.2e-16	< 2.2e-16	< 2.2e-16
Fixed effect of time (m3)	2.735e-08	1.269e-05	1.296e-13	1.968e-06
Random effect of time (random slope) (m4)	NA	1.605e-11	0.5965	0.08574
Intercept for starting work (m5)	3.824e-05	0.09084	< 2.2e-16	0.02271
Slope for starting work (m6)	0.1101	<2.2e-16	1.324e-06	0.02197
Squared overall slope (timeyrs2) (m7)	0.004919	0.01424	0.002943	0.008204
Squared starting work slope (timesince2) (m8)	0.009981	< 2.2e-16	0.002243	0.01849
