# Supplementary material for: New job, new habits? A multilevel interrupted time series analysis of changes in diet, physical activity and sleep among young adults starting work for the first time
Source: Int J Behav Nutr Phys Act. 2025 Jan 28;22:10. doi: 10.1186/s12966-024-01682-8 (PMC11773725; doi:10.1186/s12966-024-01682-8)
Supplement: Supplementary file 2 — Supplementary Material 2: Supplementary Table 2: Sensitivity Analysis Description: Additional file 2: Table of changes in health behaviours among participants with an exact job start date. [file 12966_2024_1682_MOESM2_ESM.docx]

*Supplementary Table 2:* Supplementary analysis of four health behaviours, only including participants with an exact job start date

|  |  | Initial slope  [95% CI],  *p-value* | Transition into work [95% CI],  *p-value* | Change in slope after starting work  [95% CI],  *p-value* |
| --- | --- | --- | --- | --- |
| Physical Activity (MET-min/day) |  | 0.48  [-1.93,2.90],  *0.694* | 180.57  [27.94, 333.20]*,*  *0.021* | -13.98  [ -94.60, 66.65],  *0.734* |
|  |  |  |  |  |
| Sleep (minutes) |  | 1.18  [-6.81,9.18],  *0.773* | -20.15  [-55.20, 14.90],  *0.261* | -1.37  [-12.53, 9.80],  *0.811* |
|  |  |  |  |  |
| Vegetable (portions) |  | 0.00  [-0.01,0.01],  *0.894* | 0.05  [-0.40, 0.50],  *0.833* | 0.00  [-0.17,0.18],  0*.969* |
|  |  |  |  |  |
| Fruit  (portions) |  | -0.01  [-0.02, 0.00],  *0.012* | 0.30  [-0.05, 0.65],  *0.097* | 0.18  [0.03, 0.33],  *0.019* |
