# Supplementary figures and images for: New job, new habits? A multilevel interrupted time series analysis of changes in diet, physical activity and sleep among young adults starting work for the first time
Source: Int J Behav Nutr Phys Act. 2025 Jan 28;22:10. doi: 10.1186/s12966-024-01682-8 (PMC11773725; doi:10.1186/s12966-024-01682-8)

*Supplementary Figure 1: Directed Acyclic Graphs of interaction models and confounders*

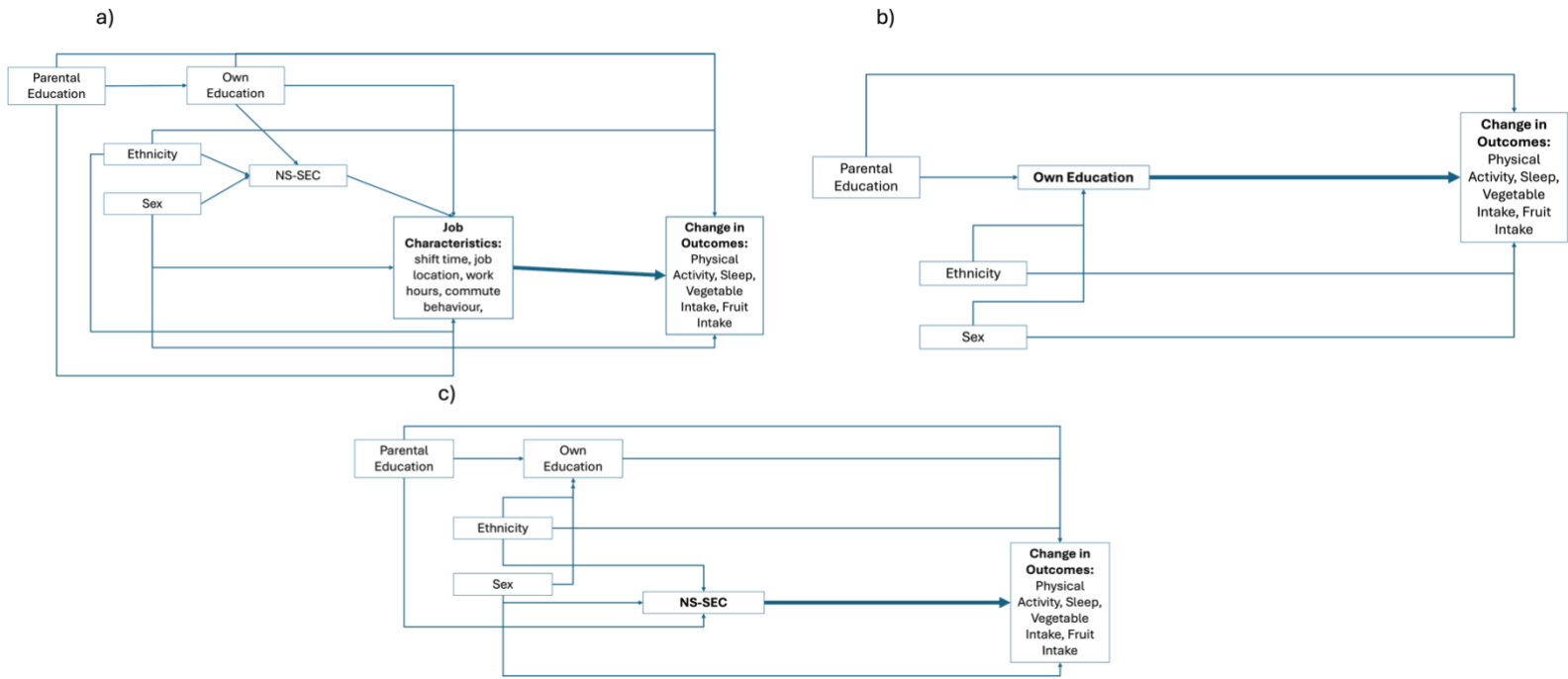

Supplement: Supplementary file 3 — Supplementary Material 3: Supplementary Figure 1: DAGs Description: Additional file 3: Directional Acyclic Graphs to show how each interaction model was adjusted for. [file 12966_2024_1682_MOESM3_ESM.pdf]
