# Supplementary material for: New job, new habits? A multilevel interrupted time series analysis of changes in diet, physical activity and sleep among young adults starting work for the first time
Source: Int J Behav Nutr Phys Act. 2025 Jan 28;22:10. doi: 10.1186/s12966-024-01682-8 (PMC11773725; doi:10.1186/s12966-024-01682-8)
Supplement: Supplementary file 5 — Supplementary Material 5: Supplementary Tables 6-9: Unadjusted models Description: Additional file 5: Tables of unadjusted interaction models for sleep, vegetable & fruit intake models. [file 12966_2024_1682_MOESM5_ESM.docx]

**Unadjusted Models**

*Supplementary Table 6*: Unadjusted Physical Activity Models. Changes in MET-min per day over time before and after starting work. Initial slope shows baseline trends per year before employment transition, Transition into work shows change in intercept immediately after starting work, and Change in slope after starting work shows longer term trends in MET-min per day each year after starting work.

|  |  | Initial slope [95% CI] | Transition into work [95% CI] | Change in slope after starting work [95% CI] |
| --- | --- | --- | --- | --- |
| University Education (n=3041) | Attended university | 12.40  [0.97,23.84] | 55.76  [13.66,97.86] | -37.33  [-54.53,-20.13] |
|  | No university | 3.94  [-13.24,21.13] | 162.20  [112.10,212.30] | -18.90  [-41.64,3.85] |
|  | *Interaction p-value* | *0.422* | *0.001* | *0.205* |
|  |  |  |  |  |
| NSSEC (n=2723) | High | 13.36[0.72, 26.00] | 17.92[-24.97, 60.82] | -18.68[-36.34,-1.03] |
|  | Low | 4.91[-12.1,21.93] | 190.04[138.91, 241.18] | -33.54[-57.34,-9.74] |
|  | *Interaction p-value* | *0.435* | *<0.001* | *0.326* |
|  |  |  |  |  |
| Work Hours  (n= 2884) | Full-time | 5.95[-6.41,18.31] | 112.17[69.17,155.18] | -27.03[-45.16,-8.9] |
|  | Part-time | 10.99[-7.33,29.3] | 115.11[61.03,169.18] | -24.48[-48.97,0.02] |
|  | *Interaction p-value* | *0.655* | *0.934* | *0.870* |
|  |  |  |  |  |
| Shift Times  (n= 2129) | Day time | 0.87[-14.28,16.01] | 113.66[65.39,161.93] | -18.71[-38.43,1.00] |
|  | Nighttime | -5.41[-73.23,62.42] | 191.97[48.82,335.13] | -28.06[-111.71,55.58] |
|  | *Interaction p-value* | *0.860* | *0.310* | *0.831* |
|  | Rotating times | 20.53[-7.75,48.80] | 87.89[-4.03,179.81] | -38.81[-79.90,2.28] |
|  | *Interaction p-value* | *0.230* | *0.627* | *0.388* |
|  |  |  |  |  |
| Commute (n= 2616) | Inactive | 17.08[2.17,32.00] | 109.62[58.96,160.28] | -34.77[-56.52,-13.01] |
|  | Active | 4.65[-11.81,21.10] | 125.52[77.09,173.96] | -27.59[-50.46,-4.72] |
|  | *Interaction p-value* | *0.273* | *0.657* | *0.656* |
|  | No Commute | 4.09[-23.35,31.54] | -117.77  [-221.95,-13.58] | 31.27[-15.81,78.34] |
|  | *Interaction p-value* | *0.415* | *<0.001* | *0.013* |
|  |  |  |  |  |
| Work Location  (n= 2846) | Office | 11.28[-0.14,22.70] | 115.12[78.52,151.72] | -30.92[-46.84,-15.01] |
|  | Home | 4.08[-23.39,31.54] | -117.8  [-221.98,-13.62] | 31.44[-15.60,78.48] |
|  | *Interaction p-value* | *0.635* | *<0.001* | *0.014* |
|  | Travel | -14.24  [-53.93,25.46] | 233.38  [103.75,363.01] | -22.34  [-75.01,30.34] |
|  | *Interaction p-value* | *0.226* | *0.085* | *0.760* |
|  |  |  |  |  |

*Supplementary Table 7:* Unadjusted Sleep Models. Changes in minutes of sleep per night over time before and after starting work. Initial slope shows baseline trends per year before employment transition, Transition into work shows change in intercept immediately after starting work, and Change in slope after starting work shows longer term trends in sleep per night each year after starting work.

|  |  | Initial slope [95% CI] | Transition into work [95% CI] | Change in slope after starting work [95% CI] |
| --- | --- | --- | --- | --- |
| University Education (n=2819) | Attended university | -3.30[-6.11,-0.48] | -6.96[-16.93,3.02] | 4.41[1.04,7.77] |
|  | No university | 3.16[-1.15,7.46] | -16.20[-27.90,-4.50] | -4.26[-8.89,0.37] |
|  | *Interaction p-value* | *0.014* | *0.239* | *0.003* |
|  |  |  |  |  |
| NSSEC (n=2549) | High | 0.01[-3.33,3.35] | -10.80[-21.40,-0.19] | 0.61[-3.12,4.34] |
|  | Low | -2.11[-6.10,1.88] | -8.45[-20.13,3.23] | 1.62[-2.89,6.13] |
|  | *Interaction p-value* | *0.426* | *0.771* | *0.735* |
|  |  |  |  |  |
| Work Hours  (n= 2688) | Full-time | -0.59[-3.40,2.22] | -12.79[-21.88,-3.69] | 1.16[-2.10,4.42] |
|  | Part-time | -0.77[-5.68,4.14] | -7.65[-21.8,6.51] | -0.07[-5.45,5.30] |
|  | *Interaction p-value* | *0.951* | *0.549* | *0.701* |
|  |  |  |  |  |
| Shift Times  (n= 2036) | Day time | 1.98[-1.93,5.90] | -18.5[-29.63,-7.37] | -1.44[-5.73,2.86] |
|  | Nighttime | 1.60[-11.19,14.40] | -8.37[-42.83,26.10] | -1.72[-15.85,12.41] |
|  | *Interaction p-value* | *0.955* | *0.583* | *0.970* |
|  | Rotating times | -0.10[-5.22,5.02] | -9.72[-26.71,7.26] | -0.49[-6.92,5.94] |
|  | *Interaction p-value* | *0.525* | *0.397* | *0.810* |
|  |  |  |  |  |
| Commute (n=2447) | Inactive | -1.09[-4.52,2.34] | -11.78[-22.58,-0.97] | 0.53[-3.35,4.41] |
|  | Active | -0.81[-4.87,3.24] | -10.52[-22.73,1.70] | 1.81[-2.91,6.53] |
|  | *Interaction p-value* | *0.918* | *0.880* | *0.682* |
|  | No Commute | 1.35[-6.61,9.31] | -14.76[-48.18,18.66] | -6.51[-18.40,5.37] |
|  | *Interaction p-value* | *0.581* | *0.868* | *0.270* |
|  |  |  |  |  |
| Work Location  (n= 2658) | Office | -1.08[-3.82,1.66] | -8.77[-17.09,-0.44] | 1.25[-1.85,4.34] |
|  | Home | 1.44[-6.50,9.38] | -14.97[-48.39,18.45] | -6.52[-18.37,5.32] |
|  | *Interaction p-value* | *0.557* | *0.724* | *0.214* |
|  | Travel | 7.99[-1.97,17.95] | -38.11[-70.04,-6.18] | -7.65[-18.29,2.98] |
|  | *Interaction p-value* | *0.085* | *0.081* | *0.115* |
|  |  |  |  |  |

*Supplementary Table 8:* Unadjusted Vegetable Models. Changes in vegetable portions per day over time before and after starting work. Initial slope shows baseline trends per year before employment transition, Transition into work shows change in intercept immediately after starting work, and Change in slope after starting work shows longer term trends in vegetable intake per day each year after starting work.

|  |  | Initial slope [95% CI] | Transition into work [95% CI] | Change in slope after starting work [95% CI] |
| --- | --- | --- | --- | --- |
| University Education (n=3171) | Attended university | 0.06[0.01,0.10] | 0[-0.14,0.14] | -0.06[-0.11,0.00] |
|  | No university | 0.05[0.00,0.10] | 0.02[-0.11,0.15] | -0.03[-0.09,0.02] |
|  | *Interaction p-value* | *0.851* | *0.854* | *0.550* |
|  |  |  |  |  |
| NSSEC (n= 2830) | High | 0.06[0.01,0.11] | -0.02[-0.16,0.13] | -0.06[-0.12,0.00] |
|  | Low | 0.02[-0.03,0.06] | 0.12[-0.01,0.26] | -0.02[-0.08,0.04] |
|  | *Interaction p-value* | *0.179* | *0.168* | *0.299* |
|  |  |  |  |  |
| Work Hours (n=3000) | Full-time | 0.04[0.00,0.08] | 0.03[-0.10,0.16] | -0.04[-0.09,0.01] |
|  | Part-time | 0.04[-0.01,0.09] | 0.08[-0.07,0.23] | -0.04[-0.1,0.02] |
|  | *Interaction p-value* | *0.999* | *0.628* | *0.953* |
|  |  |  |  |  |
| Shift Times (n=2192) | Day time | 0.03[-0.02,0.08] | 0.08[-0.05,0.21] | -0.02[-0.08,0.04] |
|  | Nighttime | 0.04[-0.11,0.18] | -0.23[-0.63,0.16] | 0.00[-0.16,0.17] |
|  | *Interaction p-value* | *0.951* | *0.141* | *0.825* |
|  | Rotating times | 0.06[-0.01,0.14] | 0.14[-0.1,0.38] | -0.10[-0.19,-0.01] |
|  | *Interaction p-value* | *0.441* | *0.684* | *0.137* |
|  |  |  |  |  |
| Commute (n=2717) | Inactive | 0.03[-0.01,0.08] | 0.01[-0.12,0.14] | -0.02[-0.08,0.04] |
|  | Active | 0.05[0.00,0.10] | 0.06[-0.09,0.20] | -0.05[-0.11,0.02] |
|  | *Interaction p-value* | *0.623* | *0.658* | *0.556* |
|  | No Commute | 0.02[-0.13,0.17] | 0.20[-0.33,0.73] | -0.14[-0.37,0.10] |
|  | *Interaction p-value* | *0.849* | *0.502* | *0.345* |
|  |  |  |  |  |
| Work Location (n=2963) | Office | 0.04[0.01,0.08] | 0.05[-0.06,0.15] | -0.04[-0.09,0.00] |
|  | Home | 0.02[-0.13,0.17] | 0.2[-0.33,0.73] | -0.14[-0.37,0.10] |
|  | *Interaction p-value* | *0.760* | *0.582* | *0.445* |
|  | Travel | 0.04[-0.06,0.14] | 0.01[-0.29,0.30] | 0.06[-0.07,0.19] |
|  | *Interaction p-value* | *0.964* | *0.812* | *0.142* |
|  |  |  |  |  |

*Supplementary Table 9:* Unadjusted Fruit Models. Changes in fruit portions per day over time before and after starting work. Initial slope shows baseline trends per year before employment transition, Transition into work shows change in intercept immediately after starting work, and Change in slope after starting work shows longer term trends in fruit intake per day each year after starting work.

|  |  | Initial slope [95% CI] | Transition into work [95% CI] | Change in slope after starting work [95% CI] |
| --- | --- | --- | --- | --- |
| University Education (n=3177) | Attended university | -0.05[-0.08,-0.01] | 0.07[-0.05,0.18] | 0.00[-0.05,0.05] |
|  | No university | -0.02[-0.07,0.03] | -0.11[-0.22,0.00] | 0.04[-0.02,0.09] |
|  | *Interaction p-value* | *0.391* | *0.028* | *0.301* |
|  |  |  |  |  |
| NSSEC (n=2834) | High | -0.04[-0.08,0.01] | 0.02[-0.1,0.14] | 0.00[-0.05,0.06] |
|  | Low | -0.04[-0.09,0.00] | -0.03[-0.15,0.08] | 0.05[-0.01,0.10] |
|  | *Interaction p-value* | 0.898 | 0.543 | 0.262 |
|  |  |  |  |  |
| Work Hours (n=3004) | Full-time | -0.04[-0.08,0.00] | 0.01[-0.10,0.11] | 0.03[-0.02,0.08] |
|  | Part-time | -0.05[-0.10,0.01] | -0.06[-0.19,0.07] | 0.04[-0.02,0.10] |
|  | *Interaction p-value* | 0.875 | 0.439 | 0.770 |
|  |  |  |  |  |
| Shift Times (n= 2196) | Day time | -0.02[-0.07,0.02] | 0.01[-0.10,0.12] | 0.00[-0.05,0.05] |
|  | Nighttime | 0.01[-0.11,0.12] | -0.16[-0.50,0.17] | 0[-0.15,0.15] |
|  | *Interaction p-value* | *0.625* | *0.335* | *0.966* |
|  | Rotating times | -0.06[-0.14,0.03] | -0.10[-0.32,0.12] | 0.05[-0.04,0.15] |
|  | *Interaction p-value* | *0.472* | *0.392* | *0.338* |
|  |  |  |  |  |
| Commute (n=2724) | Inactive | -0.04[-0.08,0.01] | -0.02[-0.13,0.10] | 0.03[-0.02,0.09] |
|  | Active | -0.04[-0.09,0.01] | -0.05[-0.18,0.09] | 0.02[-0.03,0.08] |
|  | *Interaction p-value* | *0.923* | *0.739* | *0.773* |
|  | No Commute | -0.03[-0.15,0.08] | 0.1[-0.32,0.51] | -0.06[-0.26,0.13] |
|  | *Interaction p-value* | *0.944* | *0.612* | *0.33* |
|  |  |  |  |  |
| Work Location (2969) | Office | -0.04[-0.07,0.00] | -0.04[-0.12,0.05] | 0.03[-0.01,0.07] |
|  | Home | -0.03[-0.15,0.08] | 0.10[-0.32,0.51] | -0.06[-0.26,0.13] |
|  | *Interaction p-value* | *0.953* | *0.538* | *0.331* |
|  | Travel | -0.09[-0.22,0.04] | 0.14[-0.17,0.44] | 0.05[-0.09,0.19] |
|  | *Interaction p-value* | *0.417* | *0.281* | *0.833* |
|  |  |  |  |  |
